# Immunomodulation of NK Cells by Ionizing Radiation

**DOI:** 10.3389/fonc.2020.00874

**Published:** 2020-06-16

**Authors:** Jiarui Chen, Xingyu Liu, Zihang Zeng, Jiali Li, Yuan Luo, Wenjie Sun, Yan Gong, Junhong Zhang, Qiuji Wu, Conghua Xie

**Affiliations:** ^1^Department of Radiation and Medical Oncology, Zhongnan Hospital of Wuhan University, Wuhan, China; ^2^Department of Biological Repositories, Zhongnan Hospital of Wuhan University, Wuhan, China; ^3^Human Genetics Resource Preservation Center of Hubei Province, Human Genetics Resource Preservation Center of Wuhan University, Zhongnan Hospital of Wuhan University, Wuhan, China; ^4^Hubei Key Laboratory of Tumor Biological Behaviors, Zhongnan Hospital of Wuhan University, Wuhan, China; ^5^Hubei Cancer Clinical Study Center, Zhongnan Hospital of Wuhan University, Wuhan, China

**Keywords:** NK cell, ionizing radiation, tumor, immune response, immunotherapy

## Abstract

Natural killer (NK) cells play a critical role in the antitumor immunity. Ionizing radiation (IR) has a pronounced effect on modifying NK cell biology, while the molecular mechanisms remain elusive. In this review, we briefly introduce the anti-tumor activity of NK cells and summarize the impact of IR on NK cells both directly and indirectly. On one hand, low-dose ionizing radiation (LDIR) activates NK functions while high-dose ionizing radiation (HDIR) is likely to partially impair NK functions, which can be reversed by interleukin (IL)-2 pretreatment. On the other hand, NK functions may be adjusted by other immune cells and the alternated malignant cell immunogenicity under the settings of IR. Various immune cells, such as the tumor-associated macrophage (TAM), dendritic cell (DC), regulatory T cell (Treg), myeloid-derived suppressor cell (MDSC), and tumor exhibited ligands, such as the natural killer group 2 member D ligand (NKG2DL), natural cytotoxicity receptors (NCR) ligand, TNF-related apoptosis-inducing ligand-receptor (TRAIL-R), and FAS, have been involved in this process. Better understanding the molecular basis is a promising way in which to augment NK-cell-based antitumor immunity in combination with IR.

## Introduction

Natural killer (NK) cells, belonging to the innate lymphoid cells (1), are important effectors of tumor immunosurveillance. They play a major role in recognizing and killing tumor cells as well as secreting chemokines and cytokines to regulate adaptive immune response (2). NK cells are phenotypically defined by the expression of CD56 and the lack of CD3 (3) and can classically be divided into two populations: a CD56^bright^ CD16^dim^ population and a CD56^dim^ CD16^bright^ population. The CD56^bright^ CD16^dim^ population are immature cells, characterized by cytokines production and immunoregulation; CD56^dim^ CD16^bright^ population are mature, specialized in perforin release and target killing, and are also capable of antibody-dependent cellular cytotoxicity (ADCC) (4).

## Anti-Tumor Activity of NK Cells

NK cell functions are determined by a balance between the activity of the activating and inhibitory receptors, which are tightly regulated by transcriptional programs and post-transcriptional regulations (5). The activating receptors, notably the natural cytotoxicity receptors (NCRs), including NKp46 (6), NKp30 (7), NKp44 (8), natural killer group 2 member D (NKG2D) (9), DNAX accessory molecule-1 (DNAM-1), and CD244, recognize ligands expressed on tumor cells. For signaling, NCRs stimulate the phosphorylation of immunoreceptor tyrosine-based activation motifs (ITAMs) that engage with adaptor proteins, giving rise to downstream signaling propagation, manifested by phosphoinositide 3-kinase (PI3K) and VAV-2/3 (10) activation. NKG2D assembles with the adaptor molecule DANX activation protein 10 (DAP10), resulting in the phosphoinositide 3-kinase/protein kinase B/mammalian target of rapamycin (PI3K/AKT/mTOR) signaling cascade stimulation through phosphorylating the cytoplasmic Y-X-X-M motif of DAP10 (11). On the other hand, DNAM-1 couples with the lymphocyte function-associated antigen 1 (LFA-1), ultimately contributing to the activation of extracellular regulated kinase (ERK)/AKT (12). Initiated by the engagement of 2B4, the receptor CD244 is transferred to the lipid raft, paralleled with cytoplasmic immunoreceptor tyrosine-based switch motifs (ITSMs) phosphorylation, triggering the interaction with SLAM-associated protein (SAP), SH3-binding protein 2 (3BP2), and other signaling molecules (13). Consequently, the downstream signaling cascades bring about a strong NK cell activation via calcium mobilization, degranulation and effector proteins, and interferon (IFN)-γ and granulocyte macrophage colony-stimulating factor (GM-CSF) secretion (10–13). The inhibitory receptors, including the killer cell immunoglobulin-like receptors (KIRs) (14) and the natural killer group 2 member A (NKG2A) (15), associate with major histocompatibility complex class I (MHC I) molecules expressed on target cells and initiate immunoreceptor tyrosine-based inhibitory motif (ITIM)-mediated inhibitory signaling pathway (16).

NK cells can directly eliminate tumor cells through multiple ways: first, NK cells release cytotoxic granules containing perforin and granzymes, leading to cell lysis (2, 17). Meanwhile, the production of cytokines such as IFN-γ (18) and tumor necrosis factor (TNF)-α (19) augments NK cell cytotoxicity. Second, NK cells express TNF family members such as the TNF-related apoptosis-inducing ligand (TRAIL) and FAS ligand (FASL), which interact with their receptors respectively and elicit tumor cell apoptosis (20–22). In addition, ADCC, triggered by Fcγ receptor CD16, enables NK cell degranulation against target cells (23).

## NK Cell Exhaustion in TME

The tumor microenvironment (TME), however, usually brings about NK cell exhaustion. Dysregulation of checkpoint ligands, suppressive immune cells, such as the regulatory T cell (Treg), myeloid-derived suppressor cell (MDSC), and tumor-associated macrophage (TAM), tumor-associated exosomes, transforming growth factor (TGF)-β cytokine secretion, and hypoxia might restrict NK cell function in assorted ways while the molecular mechanisms remain largely undiscovered (15).

## Modulation of NK Cell Activity by IR

Ionizing radiation (IR), one of the key cornerstones in cancer treatment, causes double-strand DNA breaks, contributing to tumor cell death. Moreover, IR elicits specific molecular changes sensed by the immune system either within tumors or in normal tissues, rendering tumor cells either more susceptible or more tolerant to immune attack (24). It has previously been demonstrated that IR boosts the immune response by augmenting the antigenicity and the adjuvanticity of malignant cells and by interacting with the TME (25). Meanwhile, IR can mediate robust immunosuppressive effects through altering particular ligands [e.g., programmed death ligand 1 (PD-L1) (26, 27), NKG2D (28)], secreting suppressive cytokines [e.g., TGF-β (29), interleukin (IL)-6 (30), and IL-10 (31)], surging the recruitment of immunosuppressive cells [e.g., Tregs (31), MDSCs (32), and TAMs (30)] and aggravating hypoxic TME (33).

**Figure 1:**
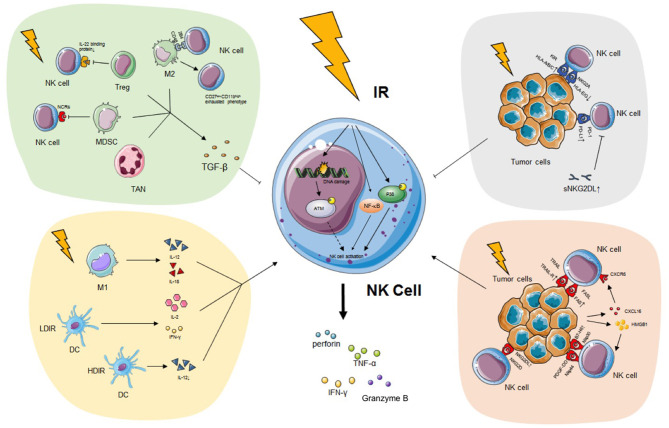
The impact of IR on NK cell. IR has a pronounced effect on modifying NK cell biology both directly and indirectly. On the one hand, IR induces the secretion of IFN-γ, TNF-α, perforin, and granzyme B of NK cells possibly through the p38-MAPK, ATM, and NF-κB pathway without alteration of activating receptors. On the other hand, IR programs the differentiation of classical activated macrophage (M1), which releases immunostimulatory IL-12 or IL-18 and triggers NK cytotoxicity. DC exposed to LDIR produces enhanced IL-2 and IFN-γ which promote NK functions while DC exposed to HDIR secretes less IL-12. IR also leads to the recruitment and activation of pro-tumor TAN phenotype (N2), alternatively activated macrophage (M2), Treg and MDSC which secrete TGF-β and impair NK activity. Finally, tumor expressed ligands, such as NKG2DL, TRAIL-R, and FAS, are upregulated during IR, enhancing the recognition of malignant cells by NK cells. However, PD-L1, classical HLA class I, sNKG2DL are also upregulated during IR, impairing the immunogenicity of tumor cells and NK cell recognition. See the main text for details. IR, ionizing radiation; NK cell, natural killer cell; IFN-γ, interferon-γ; TNF-α, tumor necrosis factor-α; MAPK, mitogen-activated protein kinase; ATM, ataxia telangiectasia mutated; NF-κB, nuclear factor kappa B; IL, interleukin; DC, dendritic cell; LDIR, low-dose ionizing radiation; HDIR, high-dose ionizing radiation; TAN, tumor-associated neutrophil; Treg, regulatory T cell; MDSC, myeloid-derived suppressor cell; TGF-β, transforming growth factor-β; NKG2DL, natural killer group 2 member D ligand; TRAIL-R, TNF-related apoptosis-inducing ligand-receptor; PD-L1, programmed death ligand 1; HLA, human leukocyte antigen; sNKG2DL, soluble natural killer group 2 member D ligand.

The impacts of IR on immune cells, such T cells, macrophages, and dendritic cells (DCs), have been extensively studied. Likewise, the effect of IR on NK cells has attracted increased attention in recent years. In this review, we summarize the impact of IR on NK cells and its potential significance in antitumoral immune response (Figure 1). It could be a promising strategy to enhance NK cell cytotoxicity and reverse NK cell exhaustion.

### Direct Regulation of NK Cell Functions by IR

High-dose ionizing radiation (HDIR) is detrimental, causing cell apoptosis, while low-dose ionizing radiation (LDIR) can benefit living organisms via stimulating immune competence (34, 35). *In vitro* LDIR at 75–150 mGy was observed to have a most pronounced effect on expansion and secretion of NK cell effector proteins, such as IFN-γ and TNF-α, possibly through the p38-mitogen-activated protein kinase (MAPK) pathway, which could be visibly potentiated by low dose of pre-radiation IL-2 treatment (36, 37). Alteration of activating receptors are not observed when NK cells undergo LDIR, suggesting that an independent regulation of NK cell cytotoxicity is mainly due to intrinsic cell proliferation and effector protein expression. Similar results have been obtained with tumor-bearing rats exposed to low-dose total-body irradiation (TBI) (0.1 or 0.2 Gy X rays), leading to the suppression of experimental tumor metastases along with the stimulation of NK cell cytolytic functions post-irradiation (38, 39). Moreover, it has also been reported that levels of phosphorylation of ataxia telangiectasia mutated (ATM), a marker of DNA damage response, increased during NK cell activation, indicating IR might regulate NK function through the DNA damage pathway (40). The nuclear factor kappa B (NF-B) signaling activation initiated by IR may exert a positive potential on granzyme B gene transcription as well as perforin gene expression (41, 42) and autophagy triggered by IR holds a decisive place in NK cell differentiation (43). However, the superb irradiation doses motivating these NK cell functions remain unearthed.

As in the case of HDIR (single dose ≥1.0 Gy), although NK cells showed partially impaired functions (44), IL-2 pretreated NK cells were more radioresistant, with their cytotoxicity being not abrogated following 30 Gy IR (45). Fractionated irradiation, 15 Gy × 2 applied at diverse intervals as well as 2.5 Gy × 4 applied at the same intervals, resulted in elevations of adenosine triphosphate (ATP) level and NK cell cytotoxicity compared to single irradiated controls delivered with 30 Gy and 10 Gy, which suggested that fractionated irradiation might be conducive to maintain NK cell functions as compared to single-dose irradiation, with the mechanism remaining uncovered (46). Taken together, the regulation of NK cell functions by ionizing radiation is strongly affected by the irradiation doses. LDIR tends to stimulate the NK cell cytotoxicity, and HDIR, especially the single-dose irradiation, is more likely to undermine the NK function, which can be reversed by IL-2 pretreatment. While optimal fraction schemes, IL-2 pretreatment and irradiation doses that are favorable to NK functions remain to be determined, the research into molecular mechanisms will with no doubt promote the utilization of NK cell-derived therapies in cancer.

### NK Cell Function Adjusted by Other Immune Cells

#### Tumor-Associated Macrophages (TAMs)

Macrophages are highly plastic cells that can be polarized toward classically activated phenotype (M1) and alternatively activated phenotype (M2). M1-like macrophages perform a dominant role in fighting against bacterial infections and malignant tumors while M2-like macrophages are proficient effectors in tissue remodeling, angiogenesis, immune regulation, and tumor progression (47). LDIR (doses ≤2.0 Gy) programmed TAMs toward an M1-like phenotype (48, 49) characterized by immunostimulatory IL-12 or IL-18 release and NF-κB pathway activation (50, 51), triggering cytolytic NK cell function (52). On the contrary, HDIR (doses ≥2.0 Gy) promoted M2-like phenotype activation (53–55). In this setting, M2-derived TGF-β decreased tumor infiltrating NK expression of Ki-67 as well as secretion of IFN-γ and TNF-α (56). Furthermore, M2 induced a CD27^low^CD11b^high^ exhausted NK cell phenotype (57). Finally, tumor-associated macrophages expressed higher levels of CD48, mediating transient activation and subsequent dysfunction of NK cells via CD48-2B4 interactions (58). Therefore, IR may indirectly regulate NK cell activity via shaping the phenotypes of TAMs, and we may bring about promising anti-tumor effect of NK cells by combining IR with M2 population reversion.

#### Regulatory T Cells (Tregs)

Tregs are observed to maintain immune tolerance and hinder suppression of infections and cancers by inhibiting effector T cells, B cells and NK cells (59, 60). The capacity of IR to induce TGF-β signaling (61) and vascular endothelial growth factor A (VEGFA) secretion (62) in the tumor environment favored the expansion of Treg cells. For instance, a local tumor irradiation of 15 Gy promoted Treg accumulation in C57BL/6 mice bearing B16-OVA murine melanoma while fractionated 2 × 7.5 Gy and 3 × 5 Gy maintained lower Treg numbers (63). Likewise, 2 Gy γ-radiation TBI significantly increased the frequency of Treg cells in peripheral blood, lymph nodes, spleens, and thymus in mice (64). However, the systemic surge of Tregs has been observed to suppress NK-cell-mediated cytotoxicity through reducing IL-22 binding protein that is known to inhibit tumor cell proliferation (65). Moreover, Tregs selectively expressed membrane-bound TGF-β, downregulating NKG2D expression and impairing NK-cell-mediated tumor immunosurveillance (66). Finally, Tregs augmented endonuclease G (EndoG) expression and led to NK cell senescence as a consequence of telomerase reverse transcriptase (TERT) mRNA alternative splicing and telomerase inhibition (67). Thus, the application of chemopreventive drugs makes it possible to block Tregs at an earlier stage and to preserve activity of NK cells.

#### Myeloid-Derived Suppressor Cells (MDSCs)

MDSCs contribute to the immune escape of pathogens and malignant tumors by restraining T cells and NK cells as well as maturation of DCs (68). IR boosted the secretion of IL-6 (69) and colony stimulating factor 1 (CSF1) (32) from irradiated cells, mediating robust chemotactic effects on MDSCs. A systemic increase of CD11b^+^Gr-1^+^ MDSCs had been documented in the spleen, lung, lymph nodes, and peripheral blood when RM-1 and Myc-Cap implanted C57BL/6 mice were exposed to 5 × 5 Gy hypofractionated radiotherapy (RT) depending on CSF1 pathway (32). Likewise, non-small cell lung cancer patients treated with RT (2.75 Gy in 24 fractions) preceding by low-dose cisplatin were intensively infiltrated with CD14^+^HLA-DR^low^ and CD14^+^CD33^+^HLA-DR^low^ MDSCs (70). However, MDSCs have been observed to induce anergy of NK cells via membrane-bound TGF-β and interaction with the NKp30 receptor (71–73). Similar results have been obtained with cancer-expanded MDSCs in a liver cancer-bearing murine model where NK cell functions were exacerbated, exhibiting as reduced degranulated cytotoxicity, NKG2D expression and IFN-γ production both *in vitro* and *in vivo* (72). Finally, CD247, a key subunit of NCRs and CD16, was observed to be downregulated on the NK surface by MDSCs (74). Inspired by the previous findings, inhibiting the infiltration of MDSCs to TME as well as targeting MDSC-NK crosstalk may abrogate NK anergy and reject tumor progression more effectively.

#### Dendritic Cells (DCs)

DCs are the most potent antigen-presenting cells that prime a T cell response and regulate both the innate and adaptive immunity (75). Previous studies have demonstrated that LDIR (doses ≤0.2 Gy) significantly enhanced IL-2 and IFN-γ production of DCs through ATM/NF-κB cascade (76, 77), stimulating NK cell survival, proliferation and cytotoxicity (78). On the other hand, mature dendritic cells exposed to HDIR (with doses ranging from 2 to 30 Gy) secreted less IL-12 (79), an essential contributor to perforin-mediated NK cell cytotoxicity and IFN-γ production (80). Regarding these supreme investigations, we believe that LDIR is able to enforce NK-DC interaction, leading to more efficient tumor recognition and retardation by NK cells.

#### Tumor-Associated Neutrophils (TANs)

Neutrophils are the first line of defense against infection and inflammation while TANs tend to exert protumoral functions (81). IR-induced TGF-β signaling in the tumor microenvironment led to the recruitment and activation of TANs with a pro-tumor phenotype (82), paralleled by facilitating NK cell dysfunction and preventing NK cells from mediating clearance of tumor cells (83), which can be overcome by the blockade of TGF-β.

To conclude, Tregs, MDSCs and TANs are likely to induce NK cell anergy when exposed to IR. TAMs and DCs, under the circumstance of LDIR, are inclined to activate NK cell functions, which will otherwise impair NK activity when treated with HDIR (Table 1).

**Table 1:** Comparisons of LDIR and HDIR in response.

**Responses**	**LDIR**	**Dose ranges**	**HDIR**	**Dose ranges**
Direct effect on NK cells	NK cell activation (36, 37) Tumor metastases suppression (38, 39)	≤ 0.2 Gy	NK cell cytotoxicity impaired (44, 45)	≥1.0 Gy
TAM-NK interaction	M1 differentiation, IL-12, IL-18 release (48–51) NK cell activation (52)	≤ 2.0 Gy	M2 recruitment, TGF-β secretion, CD48 expression (53–56) NK cell dysfunction (57, 58)	≥2.0 Gy
DC-NK interaction	IL-2, IFN-γ secretion by DCs (76, 77) NK cell activation (78)	≤ 0.2 Gy	IL-12 less production by DCs (79) NK cell cytotoxicity impaired (80)	2-30 Gy

*LDIR, low-dose ionizing radiation; HDIR, high-dose ionizing radiation; NK cells, natural killer cells; TAM, tumor-associated macrophage; IL, interleukin; TGF-β, transforming growth factor-β; DC, dendritic cell; IFN-γ, interferon-γ.

### Immunoediting of Immunogenic Malignant Cells

The recognition of malignant cells by NK cells could be altered after exposure to IR, partially through the modulation of tumor exhibited ligands, thus affecting malignant cell immunogenicity.

#### NKG2D Ligands

NKG2D is a major activating receptor expressed on all NK cells, and NKG2D ligands are usually restricted or deficient on normal tissues to escape autoimmune injury (84). *In vitro*, IR significantly upregulated NKG2D ligands in a time-dependent manner, rendering malignant cells more sensitive to NK cells (28, 85), which was probably ascribed to the DNA damage response pathway (86). However, discordances in NKG2D mRNA and surface protein expression levels were observed, reflecting differential transcriptional and post-transcriptional regulation of NKG2D expression (28). In line with these possibilities, histone deacetylase (HDAC) inhibitors, modifying the post-transcriptional epigenetic, enhanced IR-induced NKG2D ligand expression and increased susceptibility of tumor cells to NK cells. *In vivo*, IR gave rise to survival benefit in glioma-bearing mice, which could be abrogated by an anti-NKG2D antibody (87). To summarize, IR modulates the expression of NKG2DLs in time- and dose- dependent manners, which could be targeted to enhance NK cell tumoricidal effect.

In addition to NKG2DL, a shed form of NKG2DL, s-NKG2DL, generally serves as an immunity inhibitor (88), binding to the NKG2D receptors and impairing NKG2D function (89). IR increased the expression of matrix metalloproteinase-2 (MMP2) and a disintegrin and metalloproteinase domain-containing protein-10 (ADAM10) in a dose-dependent manner, mediating proteolytic shedding of NKG2D. Altogether, IR induces the surface expression of NKG2DL as well as the shed form of NKG2DL, s-NKG2DL, efficiently, and the latter suppresses NK cell function, which could be reversed by the combination of IR and MMP inhibitors (90).

#### NCRs Ligands

Several tumor-associated ligands for NCRs have been observed, including galectin-3 and B7-H6 (a newly identified ligand in the B7 family) for NKp30 (91), mixed-lineage leukemia protein-5 (MLL5), proliferating cell nuclear antigen (PCNA), nidogen-1 (NID1), platelet-derived growth factor (PDGF) for NKp44 (92), and vimentin for NKp46. Galectin-3, a lately discovered β-galactoside-binding protein expressed on tumor cells (93), was described as NKp30 inhibitor, restraining NKp30-mediated NK functions and tumor escape elimination by NK cells (93, 94). Luckily, IR alone did not change galectin-3 expression (95). Another NKp30 activator, B7-H6, is widely expressed on tumor cells (96–98). Malignant cells were more sensitive to NK cell lysis as a consequence of B7-H6 upregulation in response to IR (99). Few studies concerning the effect of IR on NKp44 ligands. IR deposits onto tumor cells as a stress inducer, producing pro-angiogenic cytokines such as PDGFs. PDGFs keep endothelial cells and vessels from irradiation-derived injury and provide nourishment and oxygen for malignant cells (100). PDGF-DD, one of the dimeric isoform of the PDGF family, was recently reported to be a ligand for NKp44 and trigger NK cell activation through evoking ITAM signaling and inducing phosphorylation of AKT, ERT in human NK cells (101); yet, there are no publications detailing effect of IR on other NKp44 ligands, such as MLL5, PCNA and NID1. Several studies reported that vimentin, a NKp46 ligand, increased in a variety of malignant cells in response to IR (102, 103). However, no studies reveal alteration of NK functions arising from vimentin upregulation, making it difficult to provide a view into the IR impact on NKp46 ligands.

#### TRAIL Receptors and FAS

IR also increases death receptors such as FAS and TRAIL on tumor cells, enhancing malignant cell destruction by NK cells. Enhanced FAS with biological activity was reported on colorectal cell lines exposed to IR (with the maximum upregulation at 10–20 Gy) mainly due to the influences on epigenetic enzyme combination and histone acetylation (104, 105). Along similar lines, significant FAS induction was obtained in patients with diffuse B-cell lymphoma and early-stage breast cancer imposed to RT (106). In addition, it's reported that TRAIL receptors (DR4 and DR5) were upregulated when prostate, breast, colorectal, and lung cancer cells were exposed to IR (21, 107, 108), suggesting a potential mechanism of NK cell mediated elimination.

#### HLA

Human leukocyte antigen (HLA) molecules can be divided into two subtypes: HLA class I, constitutively expressed by all somatic cells and HLA class II, only presented on antigen-presenting immune cells. HLA class I could be subdivided into classical (including HLA-A, HLA-B, and HLA-C) and non-classical (including HLA-E, HLA-F and HLA-G) subtypes. The classical subtype is critical for NK cell inhibition and cytotoxic T cell activation. Several publications illustrated that IR yielded upregulation of classical HLA I, indicating a negative picture for NK cell activation. For instance, IR upregulated HLA I expression in multiple myeloma, glioblastoma and meningioma in a dose-dependent manner (109, 110), with a peak at 12 Gy in brain tumors. Furthermore, an incremental dose of 0, 6, 12, and 24 Gy augmented HLA class I surface expression gradually in human renal clear cell carcinoma cells (111). However, HLA-G is a strong immunosuppressive molecule binding to inhibitory receptors existed on immune cells (112, 113), contributing to NK cell and T cell dysfunction (114–116). Previous studies illustrated that surface HLA-G1 suffered a diminish in FON melanoma cells exposed to a dose of 20 Gy as well as a fractionated 20 Gy irradiation (2 Gy × 10) (117). Similar results have been obtained with HLA-G expression on basal cell carcinomas in patients (118). HLA-E was described as a ligand capable of binding to the inhibitory CD94/NKG2A receptor on NK cells. Together with HLA-G1 expression decrease, the HLA-E level was downregulated in FON melanoma cells exposed to 10 and 20 Gy (117), mainly due to an indirect influence of HLA-G1 decrease induced by IR concerning the HLA-E stabilizing ability of HLA-G1 molecule (119). In conclusion, IR results in the upregulation of surface HLA classic I molecules as well as diminution of HLA-G and HLA-E molecules on which we should take advantage of increasing tumor susceptibility to NK cells.

#### CXCL16 and HMGB1

The upregulation of C-X-C motif ligand 16 (CXCL16) in human breast cancer cells exposed to IR resulted in a systemic surge of C-C motif chemokine receptor 6 (CCR6), expressed in NK cells through CXCL16–CCR6 interaction, which was abrogated in the presence of an anti-CXCR6 Ab (120). Additionally, a 15 Gy IR caused increased high mobility group box-1 protein (HMGB1) release in B16 melanoma cell supernatant (121), enhancing INF-γ secretion from macrophage-stimulated NK cells (122).

#### PD-L1

PD-L1 immune checkpoint plays a critical role in immunodepression. The PD-1/PD-L1 pathway has been reported to modulate NK cell function versus various malignant cells *in vivo* and *in vitro* (123–125). It has been newly reported that IR increased PD-L1 expression and decreased expression of NKG2DL in radioresistant malignant cells, protecting tumor cells from NK cell cytotoxicity through the IL-6-the mitogen-activated protein kinase (MEK)/ERK pathway, which could be abrogated with the combination of PD-L1 Ab and MEK/ERK inhibitor (126).

## Clinical Research With Regard to RT Administration on NK Function

Previous reports have illustrated that RT does indeed affect the NK functional activity in cancer patients, with irradiated sites being more significant than RT doses (127–129). For instance, greater absolute NK count diminution and NK activity reduction were detected in patients who had diverse malignances delivered with IR doses ranging from 30 to 45 Gy fractionated into 15–25 schemes (single doses ranging from 1.8 to 2.5 Gy), including an area of mediastinum exposure compared with those underwent treatment with other fields, while the proportion of NK cells remained stable or even higher (128). Along similar lines, breast cancer patients receiving conventional fractionated RT at 60–65 Gy (single doses ranging from 1.8 to 2.2 Gy) exhibited lymphopenia and the loss of NK lytic function against K562 target cells immediately. In contrast with those who also received regional treatment, NK cell activity did not drop significantly in patients who received RT to breast only and resumed at the end of 6 weeks after RT (129). It is possible that circulating NK cells transit through the mediastinum and the more radiosensitive NK precursors in the lymph nodes are directly killed by RT. Therefore, in order to preserve NK cell functions, it would be preferred to avoid large vessels as well as unnecessary lymph node irradiation.

In breast cancer patients receiving adjuvant radiation therapy (single doses with 2 Gy), significant higher number of NK cells and higher ratio of IFN-γ^+^ NK cells with increased cytotoxicity were detected even at 12 months post-RT (130). Likewise, pelvic irradiation (single doses with 2 Gy) in cases of prostate cancer or seminal vesicle tumor also induced sustained proliferation of peripheral NK cells during and 3 months after RT (131). Moreover, it has been proven that the enhancement of NK proficiency facilitated the achievement of pathological complete response in cancer patients (132). These clinical observations indicate proliferation- and activation-inducing roles of RT on NK cells, providing cues of NK cell contributions to an enduring anti-tumor immune response evoked by RT.

Although a number of studies demonstrated conventional fractionated RT promoted NK cell proliferation and activation, hypofractionated RT showed contradictory results. For example, blood samples of oligometastatic breast cancer patients received stereotactic body radiotherapy (SBRT) (30 Gy in 3 fractions) to the metastatic lesions showed elevated number and activation of NK cells characterized by nuclear translocation of NF-B 24 h after the first dose (133). Other publications have shed light on the impact of SBRT on lung cancer. A detectable augmentation of CD56^high^ CD16^+^ NK cell frequency persisting throughout 6 months without statistically importance compared to baseline was observed in blood samples of lung cancer patients administrated with SBRT (7.5 Gy × 8 or 12.5 Gy × 4) (134). Quite the reverse occured, as another clinical research enrolling a larger cohort of lung cancer patients received a total dose ranging from 40 to 70 Gy fractionated into 4–10 days (single doses ranging from 6 Gy to 15 Gy, with most patients received single doses ranging from 10 Gy to 15 Gy) revealed decreased NK counts and activity immediately and at 1 week post-IR without the alteration of NK ratio in periphery (135). These divergent results may stem from different RT fraction doses, which have been proved to impact significantly on immune-related gene expressions (136). Thus, appropriate RT doses and schedules should be administered to elicit antitumor immunity (137).

To conclude, although HDIR seems to exert a negative effect on NK function *in vitro*, conventional fractionated RT, with no doubt, is beneficial to NK function, possibly due to the interaction between NK cells and other ingredients in the TME. In the case of SBRT, a moderate fractionated dose might be proposed for the optimal therapeutic response to both irradiated and abscopal sites. Additional factors, such as lymph node exposure, proliferative state, as well as degree of differentiation, might also occupy essential roles (138). These questions are important issues to be addressed in order to understand the radiobiology in NK cells and to determine optimal RT protocol that improves anti-tumor NK cell functions.

## Conclusion

As a supplementary for anti-tumor T cell immunity, NK cells directly kill tumors without relying on antigen presentation in a major histocompatibility complex-independent way. NK cells have become a new tool for anti-tumor immunotherapy. Promising ingressions of engineered NK cells into clinical research have been observed in various tumors. Radiation therapy, one of the cornerstones of tumor treatment, has been verified in recent years to regulate immune system function widely with the molecular basis of radiation-activated NK cells being preliminary clarified. By modifying the NK cell biology, more research is warranted to boost NK cells against cancer in combination with IR.

## Author Contributions

JC, XL, QW, and CX reviewed relevant literatures and drafted the manuscript. ZZ, JL, YL, WS, YG, and JZ analyzed and revised the manuscript. All authors read and approved the final version.

## Conflict of Interest

The authors declare that the research was conducted in the absence of any commercial or financial relationships that could be construed as a potential conflict of interest.
